# The Lost Leg of the Youth Justice and Criminal Evidence Act (1999): Special Measures and Humane Treatment

**DOI:** 10.1093/ojls/gqab014

**Published:** 2021-05-11

**Authors:** Samantha Fairclough

**Keywords:** special measures, vulnerable witnesses, vulnerable defendants, humane treatment, criminal trials, criminal evidence

## Abstract

This article examines the purpose behind the provision of special measures—adaptations to courtroom formalities—to vulnerable or intimidated people giving evidence in criminal trials. It shows that fostering the principle of humane treatment and improving evidence quality were the initial motivations underpinning the introduction of special measures. The focus in this article is on the principle of humane treatment, and whether the current provision of special measures successfully fosters it. The article conceptualises the principle of humane treatment so that it can be used as the normative measure against which to evaluate the current provision of special measures to vulnerable witnesses and the accused. It concludes that the solely instrumental legal basis on which special measures are currently available risks insufficiently protecting vulnerable or intimidated participants from the harm and distress that the criminal trial often causes, and that this can amount to inhumane treatment.

## 1. Introduction

Special measures adjust the traditional way in which witnesses give evidence in criminal trials. They permit a witness to give evidence from behind a screen so that they are out of sight of the dock and public gallery; from a room outside of the courtroom via live link; with the assistance of an intermediary (a communication specialist); or using communication aids.^1^ A special measures direction may also require the lawyers in court (including the judge) to remove their traditional court dress (wigs and gowns); for those in the public gallery to leave while a witness gives evidence in private; or for the witness’s evidence to be pre-recorded and played at the trial in their absence.^2^ The Criminal Practice Directions permit the use of any combination of these measures at one time.^3^

In England and Wales, these special measures are available to vulnerable and/or intimidated non-defendant witnesses (for the prosecution and defence) via the Youth Justice and Criminal Evidence Act 1999 (YJCEA). The YJCEA sets the parameters of the categories ‘vulnerable’ and ‘intimidated’. In brief, vulnerable witnesses are children (under 18), or adults with a physical, mental or learning disability or disorder.^4^ Intimidated witnesses are those ‘in fear or distress in connection with testifying in the proceedings’.^5^ This can result from, *inter alia*, direct threats to them from the accused or their supporters; the age or background of the witness; or the nature of the offence about which they are required to testify.^6^ If a witness meets one or more of these criteria, and the quality of their evidence (in terms of its completeness, coherence and accuracy^7^) is ‘likely to be diminished’ as a result,^8^ a special measures direction should follow. Special measures are also available, albeit more limitedly,^9^ to the accused should they choose^10^ to give evidence in their defence. This is, in part, via the YJCEA,^11^ but is predominantly through the common law.^12^ The important differences in their eligibility for special measures are discussed in section 4.

The outline of the statutory criteria for special measures above highlights that an assessment of the evidence’s prospective quality is a key part of the legal test for special measures. This article shows, through an examination of their legal development, that special measures were primarily born out of an intrinsic concern for the humane treatment of the vulnerable in court. As we will come to appreciate, these two aims of humane treatment and best evidence are undoubtedly intertwined, although it is only the latter that appears as a specific criterion in the legislation. The central focus of this article is the extent to which the provision of special measures to vulnerable or intimidated witnesses and accused persons sufficiently upholds the principle of humane treatment. In order to make this assessment, the article conceptualises the principle of humane treatment—a foundational principle of criminal evidence^13^—so that it can be used as a normative standard with which to evaluate the current provision of special measures. This article is the first to engage on a conceptual level with the principle of humane treatment in this context and to consider the special measures scheme in this way.

The article concludes that the legal provision of special measures to vulnerable or intimidated witnesses and accused persons does not uphold the principle of humane treatment as a deontological value. Instead, the legislation only affords assistance to vulnerable or intimidated witnesses and defendants giving evidence where it would otherwise be detrimental to the quality of their testimony, but not for reasons that solely relate to their humane treatment. The implication of this is that it qualifies claims that special measures ‘provide *protection* to vulnerable and intimidated witnesses’^14^ and help to ‘safeguard’ children involved in criminal proceedings.^15^ This is particularly important given that the need for and justification of special measures was largely based on the otherwise inhumane treatment that vulnerable people in criminal trials would face. It is only by viewing the resulting special measures scheme through the lens of the principle of humane treatment that we see that it fails to uphold this intrinsic value *for its own sake*, and thus fails to respond to one of the key issues which drove its creation.

This failure is more broadly significant because it highlights a gap in the protection of (vulnerable) individuals when solely instrumental concerns are the legal driving force behind available support. This is important in other criminal justice contexts in which the principle of humane treatment is relevant, for example the inadmissibility of certain types of evidence, in particular, past sexual history evidence. In a bid to remain consistent with process-based theories of procedural justice concerned with securing procedures that treat individuals fairly irrespective of any instrumental advantage that might ensue,^16^ this analysis may result in us querying the legitimacy of (aspects of) the paradigm version of the criminal trial.

The article starts with an examination of the genealogy of special measures provision, which shows the centrality of concerns for humane treatment in their legal development. Its prominence within the debates bears emphasis, as it shows that the principle of humane treatment is not an external value that this article is applying to this area of the law, but is one that was integral to its development. The conceptualisation of the principle of humane treatment follows this section, before an evaluation of the current law’s commitment to it.

## 2. Genealogy of Special Measures: Tracing Their Purpose

Special measures became a topic of legislative reform in the mid- to late 1980s.^17^ The prominent perception was that evidence from children was unreliable.^18^ Spencer notes that the rules of evidence ‘conspire[d] to ensure that child witnesses either went unheard, or if they were heard, were disbelieved’.^19^ Such rules—including those relating to witness competency and the corroboration of child evidence^20^—presented barriers to the prosecution of those alleged to have offended against children.^21^ The Criminal Justice Act 1988 relaxed these rules so that evidence from children was more readily admissible in criminal trials.^22^

The adversarial setting in which evidence is given, requiring oral evidence in court in the presence of the accused, was (and remains) problematic for children and other vulnerable witnesses. The Criminal Justice Act 1988 enabled some children to give evidence by live link.^23^ The parliamentary debates surrounding the live link provision centred on the idea that ‘children must be treated with humanity, a humane attitude, and with methods which will not disturb them’.^24^ There was a recognition that to give evidence orally, in court, in front of the accused, was extremely difficult for a child witness. The live link provision was celebrated, cross-party, as one which would spare children ‘the ordeal of testifying in court in the presence of the accused’^25^ and ‘the horrors of court proceedings’,^26^ and would ‘remove the most acute aspects of … trauma’.^27^ It was heralded as ‘a humane step forward’.^28^ It was also recognised that the provision ‘could improve the quality of evidence before the courts’^29^ and that ‘the use of a live video link in court would also be a major advantage to the prosecution of [child abuse] cases’.^30^ The emphasis in Parliament, however, was on making the ‘whole procedure … more humane’.^31^

The Pigot Committee then considered whether pre-recorded video evidence should be admissible in criminal trials in lieu of live testimony. The arguments for its admissibility in the resulting *Pigot Report* fell into two key themes. The first centred on the ‘child’s welfare’ and the second upon the ‘integrity of the evidence’.^32^ With regard to the child’s welfare, the report highlighted that ‘quite radical changes are … required if the courts are to treat children in a humane and acceptable way’.^33^ With regard to the ‘integrity of the evidence’, the Committee’s view was that video-recorded evidence would make important evidence available^34^ and would improve the quality of children’s evidence.^35^ Overall, the Committee stated that:

The difficulties which exist in bringing those who commit offences against children to justice and the damage which courts often inflict upon many children both constitute grave and hitherto intractable social problems. We think that the development of video technology gives us an opportunity to find fair and humane solutions to these.^36^

Furthermore, the Pigot Committee suggested that video-recorded evidence (and an ‘interlocutor’, now known as an ‘intermediary’, to facilitate communication) should be available to adult witnesses who would ‘be likely to suffer “an unusual and unreasonable degree of mental stress” if required to give evidence in open court’.^37^ This approach was later referred to as ‘sensible, humane, advantageous to the court and fair’.^38^

The result of this report was the introduction of pre-recorded evidence as a child’s evidence-in-chief.^39^ Its eligibility criteria were similar to that for the live link.^40^ Again, Parliament discussed it as a measure that would ‘spare children the ordeal of giving evidence [in court]’.^41^ It was also welcomed as a provision because evidence would be secured quickly, preserving the memory of the child.^42^ The House of Lords acknowledged that ‘children are more likely to tell the truth when not in the presence of the person being accused, in a reasonably friendly environment and as near to the time of the alleged incident as possible’.^43^ There were also multiple references throughout the debate to the need to avoid cases collapsing due to the distress of the child.^44^ Meeting the needs of ‘vulnerable child witnesses’ was thus recognised as favourable ‘not only for … the child but for … the court in that a child who is properly handled is more likely to give evidence of value to the court’.^45^

In the mid-1990s, attention turned to the treatment of learning disabled witnesses. They too were subject to arbitrary competency rules,^46^ which had a negative effect on decisions to prosecute.^47^ Sanders and others also found that, at trial, cases were prone to collapse due to the judge ruling key witnesses with learning disabilities as incompetent.^48^ For witnesses with learning disabilities who were competent to testify, their limited ability to adapt to the court environment was said to have a ‘deleterious effect on memory and communication, and increase levels of stress’.^49^ Sanders and others argued that many of the existing adaptations for children designed to ‘make court appearances less terrifying’ could also be appropriate for adults with learning difficulties.^50^ They concluded that such adaptations would ‘allow learning disabled witnesses to give their best evidence’ and reduce the trauma of the process.^51^ Importantly, the researchers described the need to reduce the trauma of the process as ‘an important objective in itself, regardless of the effect on [the witness’s] eventual testimony’.^52^

A second governmental inquiry into the treatment of vulnerable witnesses was commissioned in 1997, which culminated in the report *Speaking Up for Justice.*^53^ This was a partial response to ‘New’ Labour’s manifesto, which committed to provide ‘greater protection’ to various categories of witness, in recognition of the reality that ‘many adult victims and witnesses find the criminal justice process daunting and stressful’.^54^ The Working Group declared that ‘failure to recognise and compensate for inequalities between witnesses seems both inhumane (when this results in stress or trauma for the witness) and unjust’.^55^ Its recommendations thus sought to remedy these failings.^56^ The Working Group fully supported the recommendations from the *Pigot Report*—to expand the range of special measures available and those eligible to access them.

With regards to eligibility, the Working Group identified two categories of witness: Category A and Category B. Category A witnesses were those who were vulnerable as a result of ‘personal characteristics’ (such as a significant impairment of intelligence or social functioning; a physical, mental or learning disability). The Working Group recommended that these witnesses should automatically attract the provision of special measures.^57^ Category B witnesses were those who were vulnerable as a result of particular circumstances (such as the nature of the offence; the dangerousness of the defendant; or the witness’s age, cultural background or relationship to a party in the proceeding). The Working Group recommended that the court retain discretion over the availability of special measures for these witnesses depending on whether they ‘would be likely to suffer … emotional trauma’ or ‘would be likely to be so intimidated or distressed as to be unable to give best evidence’.^58^

To an extent, these recommendations shaped the Youth Justice and Criminal Evidence Bill 1998.^59^ The Bill was discussed at length in Parliament. Familiar concerns for the treatment of vulnerable witnesses were rehashed, with widespread acknowledgement of the damage that the traditional court process inflicts on such individuals. More prominent in this round of debates was the aim of securing best evidence.^60^ For instance, the aims of special measures provisions were announced as ‘to encourage witnesses to come to court to give evidence and to help those who need it to give their evidence when they get there’.^61^

Tracing the development of the current special measures scheme shows us that two core sets of (related) concerns led to their inception and expansion. These were:

1. The lack of *protection* of children and other vulnerable people in criminal trials and the resulting trauma and damage inflicted, often amounting to *inhumane treatment*.
2. The system’s *inability to convict* those offending against children and other vulnerable people due to the lack of admissible evidence of *good quality*.

In short, the motivations underpinning the development of special measures were (i) the deontological value of the humane treatment of those involved as witnesses in criminal trials and (ii) the instrumental goal of improving the quality of witness evidence (also referred to as ‘achieving best evidence’). The bifurcation of these motivations does not mean that there are not connections between the two, as is explored later in the article.^62^ This historical account shows us that it was initial concerns for humane treatment that drove reform in this area. The focus in this article is on the law’s commitment to the principle of humane treatment.^63^

Before the current provision of special measures in the YJCEA is considered, it is necessary to turn our attention to the meaning of (the principle of) humane treatment. To establish the importance of it, and to assess whether special measures successfully foster it, the principle requires some conceptualisation.

## 3. Conceptualising the Principle of Humane Treatment

The principle of humane treatment is one of Roberts and Zuckerman’s five foundational principles of criminal evidence.^64^ It demands that ‘government in all its forms should respect the inherent dignity and exhibit appropriate regard for the welfare of every person over whom it exercises jurisdiction’.^65^ Roberts and Zuckerman argue that these five principles are the ‘moral touchstones’ against which criminal justice reforms and decisions should be made.

Roberts and Zuckerman see the principle of humane treatment as one which seeks to ensure that the accused and other witnesses in criminal proceedings are treated as:

Thinking, feeling, human subjects of official concern and respect, who are entitled to be given the opportunity to play an active part in procedures with a direct and possibly catastrophic impact on their welfare, rather than as objects of state control to be manipulated for the greater good …^66^

They locate the basis for this principle in Kant’s dignity^67^ principle: ‘Humanity itself is a dignity: for a human being cannot be used merely as a means by any human being (either by others or even by himself) but must always be used at the same time as an end.’^68^

Roberts fleshes out the principle of humane treatment further in the context of the criminal trial. He argues that, in such proceedings, ‘the dignity principle mandates that every person appearing in a courtroom should be recognised and respected as a participating subject, rather than being objectified as a means to penal ends’.^69^ This, he continues, is ‘distilled into the principle of humane treatment, a normative standard with significant traction on the administration of criminal proceedings’.^70^ Where the accused is concerned, procedural rules, which protect suspects from physical or psychological abuse, along with evidential rules that exclude unfairly prejudicial or illegally obtained evidence, all foster humane treatment.^71^ The state pursues the humane treatment of complainants and other witnesses through rules of evidence and procedure that limit the extent of cross-examination and adjust the environment in which a vulnerable or intimidated witness gives evidence.^72^ Indeed, Roberts notes that the provision of special measures to vulnerable and/or intimidated witnesses is an evidential device ‘in partial fulfilment of the principle of humane treatment’.^73^

The context in which we are concerned about humane treatment is the particular point at which an individual gives evidence in a criminal trial. Our starting point has to be to acknowledge that, within the parameters of our established system of adversarial justice, it is inevitable that witnesses and defendants will experience some discomfort when testifying. The courts are arguably ‘supposed to be daunting places in which participants are encouraged to reflect on the gravity of law and proceedings’.^74^ Mulcahy notes that the architecture of courthouses tends to ‘convey a sense of importance or foreboding’.^75^

To give evidence in a criminal trial is often ‘alienating and stressful, particularly if [the witness] is not used to speaking before an audience’.^76^ The principle of orality requires that a person testifies under oath, in the presence of lawyers (in traditional court dress), members of the public and press, and the accused, which is often inherently daunting. Cross-examination, employed to test the strength of the evidence and the veracity of the witness, is an additional and significant cause of anxiety for many.^77^ This tradition of vigorously testing oral evidence in a stressful environment remains central to the adversarial theory of truth-finding which is embedded in criminal procedure in England and Wales.^78^ That this occurs in a public arena further displays our commitment to the principle of open justice. It is inevitable, therefore, that many witnesses and defendants will experience (at least) some distress and anxiety around their testimony. In fact, it is perhaps even by design that this is the case.

This article does not argue that the level of suffering that witnesses and defendants might ordinarily experience in the criminal trial is inhumane. Oral testimony, in open court, relating to a criminal accusation—whether as a complainant, witness or the accused—is likely to provoke a stressful response in everyone. This has to be balanced against the significance of principles such as open justice and accurate fact-finding, which are vital to upholding the legitimacy of the criminal justice process and the verdicts that ensue. Witness testimony is essential to secure convictions and thus for the criminal justice system to function effectively. Provided, therefore, that the level of suffering experienced in the criminal trial is reasonably low and, importantly, is proportionate and necessary to the aims of the criminal justice system, it should not be considered inhumane.^79^ This is in much the same way that (the prospect and reality of) sitting examinations is stressful and concerning for many students. It does not mean that to subject students to assessment by examination—and thus to some level of suffering—is generally^80^ to be considered inhumane, provided that it is proportionate and necessary to assess and record their academic ability. It is therefore assumed in this article that the mode of evaluating evidence in our adversarial system, through oral and public testimony and cross-examination, generally (though not always^81^) generates an acceptable level of stress. Against this backdrop, it cannot be the case that the rules of evidence are required to eradicate *any* suffering among the participants in a criminal trial. We are concerned with suffering that goes beyond this ‘base level’ and that is not proportionate.^82^ Conceptually speaking, then, inhumane treatment thus marks a violation of relational dignity, a form of ‘non-inherent dignity’ that requires us to treat others with due respect because of our shared ‘full-inherent dignity’ status as humans.^83^

We must now turn to consider instances where the level of suffering caused in the criminal trial exceeds that which is necessary and proportionate to accurate fact-finding.^84^ Jurisprudence pertaining to article 3 of the European Convention on Human Rights (ECHR) provides clear examples. Article 3 ECHR states that ‘no one shall be subjected to torture or to inhuman or degrading treatment or punishment’.^85^ The European Court of Human Rights (ECtHR) has defined the requisite levels of harm involved in torture, inhuman and degrading treatment (or punishment)^86^ in the key case of *Ireland v United Kingdom*.^87^ The ECtHR held that, in broad terms, inhuman treatment is treatment that causes ‘intense physical and mental suffering’.^88^ Degrading treatment is that which results in ‘feelings of fear, anguish and inferiority capable of humiliating and debasing [a person] and possibly breaking their moral resistance’.^89^ Torture is the more severe of wrongs, amounting to ‘very serious and severe suffering’.^90^

In assessments of potential article 3 breaches, the ECtHR notes that the context of the case and its circumstances are relevant to the decision. This means that whether or not ill-treatment falls within the scope of article 3, and to which degree, ‘depends on all of the circumstances of the case, such as the duration of the treatment, its physical or mental effects and, in some cases, the sex, age, and state of health of the victim’.^91^ Also relevant is the ‘nature and context of the treatment’.^92^

Treatment that is torture, inhuman or degrading (as per article 3) falls within the domain of ‘inhumane’ treatment. However, despite the fact that the terms are often used interchangeably, the concept of inhumane treatment is not synonymous with inhuman treatment. This is because, as Waldron notes, a prohibition of inhumane treatment is a more exacting standard than to prohibit inhuman treatment.^93^ At a basic level, inhuman treatment is ‘treatment that is not fit or appropriate to human beings’, while inhumane treatment involves a disregard for a person’s ‘sensibilities’.^94^ In short, all inhuman treatment is inhumane, but not all inhumane treatment is inhuman.

If all inhuman (and degrading) treatment as per article 3 is inhumane, then this gives us a solid starting point from which to flesh out the meaning of the principle of humane treatment. Violations of article 3 represent some of the most serious violations of the principle of humane treatment, but this is not a complete picture. Treatment that is deplorable, but does not reach the ‘minimum level of severity’^95^ for article 3, can also fall under the auspices of ‘inhumane’ treatment. The question remains, however, as to the kind of treatment that might constitute ‘merely inhumane’^96^ treatment. In other words, how should we quantify ill-treatment that does not violate article 3 but is not necessary or proportionate to a legitimate aim of the trial (fact-finding), and is thus inhumane?

The dissenting opinion of Judge Fitzmaurice in *Ireland v UK*^97^ can help with this. He noted the difficulties which can ensue where ‘intermediate forms of maltreatment’ fall short of the degree of severity required for article 3, and regarded the Convention as ‘defective’^98^ for failing to capture these lesser forms of ill-treatment.^99^ He described treatment falling short of article 3 as ‘ill-treatment, harsh treatment, maltreatment’ or anything that causes ‘an appreciable amount of aching, strain, discomfort, distress’.^100^ What Judge Fitzmaurice was referring to here was suffering that is significant or considerable, but not ‘sufficiently severe’ or ‘intense’ to constitute degrading or inhuman treatment. If, as this article does, we accept that *some* distress or discomfort is inevitably caused when a witness or defendant testifies in a criminal trial—and importantly that this is not inhumane—then what we are looking for is suffering which is heightened from that which one would ordinarily and reasonably experience as a witness in the adversarial proceedings. This article argues, then, that inhumane treatment constitutes that which causes at least ‘a heightened amount of suffering’ to that which is generally accepted in criminal trials. It need not (but still could) be sufficiently severe to engage article 3.

The remainder of this section seeks to set some markers for what inhumane treatment looks like in the context of giving evidence in the criminal trial. Put differently, when does giving evidence in court cause an individual a heightened amount of suffering to that which we would ordinarily expect (and accept)? The way in which a person experiences the criminal trial proceedings and testifying within them may be vastly different depending on their age, health, and the circumstances of the case in which they are testifying. This is clear in the psychological literature and was acknowledged in the parliamentary debates preceding the enactment of the special measures scheme. Indeed, Ellison and Munro note that ‘current trial processes are often liable to increase rather than ameliorate trauma amongst a broad constituency of victims and witnesses’.^101^

As with the more severe forms of ill-treatment (for article 3), this article thus argues that assessments of whether treatment is inhumane should be contextualised. This means that the ‘vulnerability or intimidation’ of a witness or accused person is relevant to assessing whether their treatment in the criminal trial has caused them a heightened amount of suffering compared to their non-vulnerable counterparts, and is thus inhumane. This is in line with a commitment to a principle of substantive equality that recognises the need to treat different people differently.^102^ Indeed, Cooper and Roberts note that ‘Already-vulnerable witnesses may be especially traumatised by the experience of testifying in court, posing the risk of egregious breaches to the duty of humane treatment’.^103^

It should be noted that an individual’s differential status as the accused or a witness is *not* of material relevance to whether the treatment they receive is inhumane. While defendants enjoy procedural rights to which other witnesses are not privy, such as the right to legal representation and their non-compellability, these rights are designed to redress the ‘adversarial deficit’^104^ and do not simultaneously eradicate the potential for inhumane treatment arising from the criminal trial itself.^105^ The analysis of (in)humane treatment thus considers the accused and non-accused together, though later takes account of important differences in the legal provision of special measures to these participants.

The case of *T v United Kingdom*^106^ helps to begin to delimit the principle of humane treatment. The case involved two 11-year-old defendants tried for and convicted of murder in the Crown Court. The defendants did not give evidence in their defence, but the treatment the boys received during their trial gave rise to several complex and overlapping issues arising in their application to the ECtHR,^107^ which remain relevant when considering the challenges of testifying. The important part for our purposes is that, under Articles 3 and 6(1) ECHR, ‘*inter alia*, in view of his youth, immaturity and state of emotional disturbance, his trial in public in an adult Crown Court constituted inhuman and degrading treatment and was unfair because he was unable to fully participate’.^108^

It warrants citing the ECtHR directly to flesh out the detail of this application and the decision made. With specific regard to article 3, the applicant stated that

the cumulative effect of the age of criminal responsibility, the accusatorial nature of the trial, the adult proceedings in a public court, the length of the trial, the jury of twelve adult strangers, the physical lay-out of the courtroom, the overwhelming presence of the media and public, the attacks by the public on the prison van which brought him to court and the disclosure of his identity, together with a number of other factors linked to his sentence … gave rise to a breach of Article 3.^109^

Importantly, the applicant noted that ‘due to the conditions in which he was put on trial … he had been severely intimidated and caused feelings of anxiety and oppression by the procedures followed’.^110^

Despite this, the ECtHR concluded that ‘even if there is evidence that proceedings such as those applied to the applicant could be expected to have a harmful effect on an eleven-year-old child’, it was not convinced that

the particular features of the trial process as applied to [T] caused, to a significant degree, suffering going beyond that which would have been engendered by any attempt of the authorities to deal with the applicant following the commission by him of the offence in question.^111^

The ECtHR did not deny that the applicants had suffered, but instead concluded that the suffering was not sufficiently severe to engage article 3. Even if the ECtHR decision was correct, the applicant clearly experienced a heightened amount of suffering to that which a criminal trial would usually generate. This is because in some cases, for some individuals, factors such as their young age, the formal and public setting in which they are tried, and the presence of multiple strangers in the courtroom are such that will intensify the distress caused. As has been established, such heightened suffering is inhumane. It is this standard treatment within the adversarial context—where a defendant or a witness gives evidence in open court orally, in the presence of multiple people, subject to vigorous cross-examination, often a significant time after the alleged incident has taken place—that constitutes treatment that can be inhumane.

A further example of treatment that would constitute inhumane treatment comes from the case of *Y v Slovenia*.^112^ In this case, the accused cross-examined the applicant (the complainant) over several days with regard to allegations of sexual abuse. The ECtHR held that this amounted to a violation of her respect for private life and personal integrity under article 8 ECHR.^113^ This also amounts to inhumane treatment, in light of the fact that it caused the applicant heightened suffering that could have been avoided (and thus was not necessary).

The individual and contextual way in which people experience their treatment in the adversarial trial means that it is not possible to set an objective ‘test’ or ‘benchmark’ to measure precisely when suffering becomes so heightened as to be inhumane. The test or threshold for inhumane treatment needs an element of subjectivity to capture the effect that giving evidence in a criminal trial has on a witness or accused person. It would thus not be desirable, useful or possible to create an itemised, specific (and unwieldy) list of prohibited treatment or ‘adequate’ suffering for inhumane treatment.^114^

This leaves an inherent vagueness or uncertainty regarding the scope of the principle of humane treatment. Too much vagueness is sometimes problematic because it undermines the ability to use the law as a guide^115^ if it does not have sufficient clarity.^116^ It is important, therefore, to put down some markers to delimit when treatment will be inhumane, in order that the principle is applicable.^117^ However, it remains beneficial to leave a value-laden term such as ‘(in)humane’ somewhat vague.^118^ This is because some ambiguity may lead to criminal practitioners erring on the side of caution^119^ when considering whether adaptations should be made using special measures, rather than simply doing ‘just enough’ to avoid falling foul of some enumerated inhumane treatment threshold.^120^ This, in turn, should incentivise those working in the courts with (vulnerable or intimidated) witnesses/accused persons to be proactive in improving their treatment.

From a practical perspective, then, we can only highlight some indicators of heightened suffering, which may signal that adaptations are required to avoid inhumane treatment. Easily observable indicators of stress—signalling heightened suffering—could include seeing that a witness is visibly and excessively anxious (fidgeting, trembling, jaw-clenching suffering panic attacks, difficulty concentrating) and emotional (frequently crying, feeling withdrawn). Other, less visible manifestations could include sleepless nights, behavioural changes, a general fatigue, changed eating habits, an over-reliance on drugs and alcohol, a lack of general motivation, etc.^121^

While these more external measures might help to identify some individuals who are experiencing, or likely to experience, a heightened level of suffering as a result of testifying in a criminal trial, it will not identify all. Someone may be suffering but disguising it in their outward appearance. Rather than relying on the detection of visible manifestations of stress—which will vary for different people—a better way of assessing a witness/defendant’s well-being might be to adopt something comparable to the perceived stress scale (PSS).^122^ This would objectively measure a witness or accused person’s subjective experience of effects of the prospect and process of giving evidence in a criminal trial. The PSS comprises a series of 10 questions, the answers to which generate a ‘stress score’. Using such a scale may require its adaptation for a bespoke perceived stress scale for the criminal trial. It could help to highlight when stress and anxiety around testifying does or may amount to a heightened level of suffering and thus be inhumane. Another tool that could measure this is the state–trait anxiety inventory.^123^ Its most popular version, Form Y, asks 20 questions that measure state anxiety^124^ and 20 questions that assess trait anxiety.^125^ This approach could help to identify anxiety that is new or exacerbated by the prospect of testifying in an adversarial setting.^126^

To conclude, this article argues that inhumane treatment in the criminal trial is that which results in at least a heightened amount of suffering when compared to the standard level of discomfort that is inherent in adversarial proceedings and that is proportionate and necessary to the legitimate aim of accurate fact-finding. The principle of humane treatment, therefore, should be properly understood as a principle against treating people inhumanely.^127^ Assessments of a person’s suffering should be made contextually, with regard to the individual circumstances of the person involved (ie age, mental health issues, sex, nature of offence). It is not possible to set a standard test or benchmark to show what this level of suffering looks like in practice due to known differences in the way that people experience and react to stressful situations. Even if it were possible, this article argues that it is not desirable to set such a precise threshold for inhumane treatment, and instead that some vagueness in the law in this regard is valuable. To help in practical terms to assess when suffering may be so heightened as to be inhumane, one may be able to adopt a stress scale to measure subjective feelings of stress among vulnerable or intimidated witnesses/accused persons and make such adaptations to the traditional trial procedures as are necessary to minimise this.

## 4. Evaluating Special Measures: Does the Current Provision Uphold Humane Treatment?

Having set out the parameters of the principle of humane treatment in the context of the criminal trial, this section asks whether the current provision of special measures protects vulnerable or intimidated individuals from the heightened suffering (and thus inhumane treatment) they may otherwise experience if subjected to the same procedures as the non-vulnerable. This article does not enter into a debate as to whether the definitions of vulnerable and intimidated under the YJCEA are adequate and appropriate.^128^ Instead, the article seeks to explore whether the YJCEA provision of special measures to those it defines as vulnerable or intimidated sufficiently protects their humane treatment.

Two assumptions underpin this evaluation. The first is that a witness or defendant who is vulnerable or intimidated as per the YJCEA criteria is respectively competent to testify^129^ or fit to plead.^130^ If they are not, then they will not give evidence and thus the special measures scheme is irrelevant. The second is that so long as a person’s vulnerability or intimidation is identified^131^ and special measures are invoked, the special measures available at least have the *potential* to foster the principle of humane treatment.^132^

The focus in the first subsection is on the way in which eligibility for special measures is constructed under the YJCEA. This differs according to age and whether an individual is a witness or the accused. As is shown, the quality of the evidence elicited is the central criterion on which eligibility for special measures rests. The issue for consideration, then, becomes whether, despite this instrumental legislative focus, the current provision of special measures still safeguards the humane treatment of those giving evidence in criminal trials.

### A. Centrality of Evidence Quality

**Figure 1:**
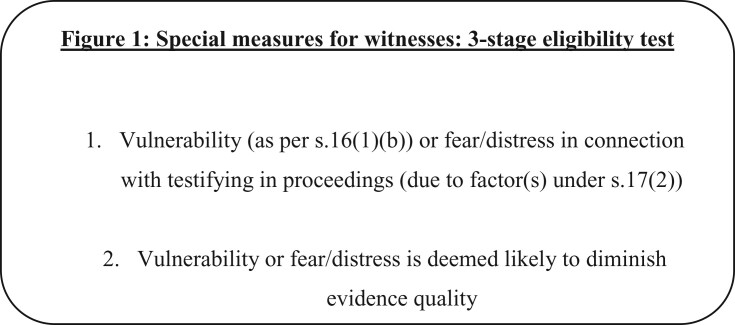
Special measures for witnesses: 3-stage eligibility test

Child witnesses^133^ automatically qualify for special measures, since the sole criterion for their eligibility is age.^134^ For vulnerable adult witnesses (over 18), there is a three-stage eligibility test (see Figure 1). First, an adult witness must have a vulnerability as per the YJCEA. This means a mental disorder as listed under the Mental Health Act 1983; a significant impairment of intelligence or social functioning; or a physical disability or disorder.^135^ Secondly, this vulnerability must risk diminishing the quality of the witness’s evidence,^136^ meaning its ‘completeness, coherence and accuracy’.^137^ The third stage of the eligibility test requires the court to determine whether the use of special measures would be likely to improve the evidence’s quality.^138^

Intimidated witnesses are those in fear or distress in connection with testifying in the proceedings.^139^ In determining eligible witnesses, there is, again, a three-stage test (see Figure 1). First, the court should consider factors such as the nature and circumstances of the offence to which the proceedings relate;^140^ the age of the witness; the behaviour of the accused or their supporters towards the witness; and the witness’s social and cultural background, ethnic origins, domestic and employment circumstances, religious beliefs and political opinions.^141^ Second, the court must consider that any fear or distress resulting from these circumstances will diminish the quality of the witness’s evidence.^142^ Third, the court must be of the view that the provision of special measures is likely to improve the quality of evidence the witness gives.^143^

What is clearly visible from these eligibility criteria for vulnerable or intimidated adult witnesses is that the legislative intent behind the provision of special measures is to improve the quality of evidence. This is an explicit part of the criteria for eligibility for a vulnerable or intimidated witness to secure special measures support. This differs from the recommendations in *Speaking Up for Justice*,^144^ which called for automatic eligibility for vulnerable adults (Category A witnesses), with no requirement for an improvement in evidence quality. It also differs from the recommendations for Category B witnesses—now intimidated witnesses. For these witnesses, special measures eligibility in the report was discretionary, but on the basis of evidence quality *or* avoidance of emotional trauma. The latter of these two points of discretion, which is really about humane treatment, did not make it into the YJCEA.

The departure from the *Speaking Up for Justice* recommendations, through the added requirement of an evidence quality criterion for vulnerable adult witnesses, was queried as the YJCE Bill progressed through Parliament. Lord Windlesham (President of Victim Support) asked the government to explain why the Bill did not follow the ‘approach proposed by the working party of their own officials’ but instead adopted ‘a far more legalistic approach, turning on the test of “improving” … or “not diminishing” the quality of evidence’.^145^ Lord Williams’s response to this was that the government ‘want[s] to focus the measures on those who really need them so that they will be available only if the court considers they will improve witnesses’ evidence’.^146^ This clearly shows the government’s priority for the instrumental issue of evidence quality over the intrinsic value of the humane treatment of witnesses required to testify.

The instrumental goal of evidence quality, rather than the principle of humane treatment, is also the driving force behind the automatic provision of special measures to child witnesses. The presumption under section 21 YJCEA is that, in sexual or violent cases, any relevant recording is admitted as the child’s evidence in chief, and further evidence elicited from the child through cross-examination is done via the live link.^147^ If the child witness wishes to give evidence in an alternative way,^148^ the court may permit this *so long as it will not result in the diminution of the witness’s evidence*.^149^ What this means is that while a presumption remains that child complainants of certain offences will give evidence using particular special measures, subject to evidence quality safeguards this is rebuttable. The motivation behind the primary rule, therefore, and by extension the automatic eligibility of special measures to children, is to improve the quality of children’s evidence, rather than a recognition that adaptations are needed for their humane treatment.

Evidence quality is also at the centre of the provision of special measures to intimidated witnesses. The inclusion of those in fear or distress in connection with testifying in the YJCEA may, at first glance, seem resonant of a commitment to the principle of humane treatment. However, any such potential for this is undermined by the caveat that special measures are only to be granted on these grounds to avoid any diminution of the quality of the evidence elicited.^150^ The same can be said of the automatic provision of special measures to complainants of sexual offences under section 17(4). As with the automatic entitlement of children, there is a presumption that relevant recordings are admitted under section 27 as evidence in chief, but this does not apply if ‘the court is satisfied that … it *would not be likely to maximise the quality of the complainant’s evidence*’.^151^ Again, this firmly places the emphasis on the instrumental goal of evidence quality rather than the deontological value of humane treatment.

The provision of special measures to the accused is different to that for all other witnesses. Their initial exclusion from the 1999 Act’s special measures scheme^152^ has resulted in their eligibility for special measures developing organically via a mixture of the common law and legislative intervention, predominantly on the grounds of effective participation and equality of arms.^153^ The only special measure available to the accused by way of statutory power is the live link.^154^ Vulnerable adult defendants are those with a mental disorder or another significant impairment of intelligence and social function who are unable to participate as a witness.^155^ For vulnerable child defendants their ability to participate effectively as a witness needs to be ‘compromised by [their] level of intellectual ability or social functioning’.^156^ Central to the accused’s eligibility for special measures, then, is their ability to participate effectively as a witness in the proceedings. This involves giving their best evidence.^157^ Much like for witnesses, therefore, there is a distinct absence of eligibility on the grounds of humane treatment and instead a focus on wholly instrumental concerns.

It is clear from this analysis of the legislative provision of special measures to vulnerable or intimidated witnesses and accused persons that the sole focus of their eligibility is on enhancing evidence quality. This is despite the prominence of the concern for the humane treatment of vulnerable witnesses in the debates that culminated in this legislation. The pertinent question then becomes whether, despite this legislative focus, the current legal provision of special measures still adequately fosters the principle of humane treatment by protecting vulnerable and intimidated individuals from heightened suffering when giving evidence. This is where the nature of the relationship between the two motivations for special measures becomes relevant.

### B. Mutually Reinforcing? Evidence Quality and Humane Treatment

In a report about the implementation of special measures provisions, Cooper and Roberts note that ‘Efforts to reverse the traditional neglect of victims’ interests … are underpinned by mutually reinforcing intrinsic and instrumental considerations’.^158^ They argue that as a matter of ‘intrinsic morality, the state owes a “duty of humane treatment” to everybody who is obliged to participate in criminal proceedings’.^159^ They further note that, from an instrumental perspective, witnesses’ evidence is often crucial to successful prosecutions.^160^ If the instrumental goal of improving evidence quality and the deontological value of humane treatment are truly mutually reinforcing, as Cooper and Roberts suggest, then the statutory emphasis on evidence quality is only a matter of semantics. This is because, in this cyclical relationship, a focus on evidence quality would automatically enhance our commitment to the principle of humane treatment.

This section considers the extent to which these aims truly are mutually reinforcing. An examination of the way in which the principle of humane treatment operates in the context of special measures shows that when special measures *are* secured on evidence quality grounds, the system’s resultant commitment to the principle of humane treatment is simultaneously improved. This is because, in this regard, the principle of humane treatment can be understood to operate in two ways: (i) as underpinning the instrumental goal of improving evidence quality; and (ii) as helping to achieve the instrumental goal by improving the treatment of witnesses. This is unpacked in the following discussion.

#### (i) The principle of humane treatment underpins the instrumental goal of improving evidence quality

Enhancing the quality of evidence elicited from vulnerable or intimidated individuals has some purely instrumental rationales. From a practical perspective, the criminal justice system is simply unworkable without witness testimony. Alleged victims and prosecution witnesses are ‘gate-keepers to the mobilisation of criminal justice agencies’^161^ due to the systemic reliance on their voluntary reporting of crime and their assistance in securing convictions where it is required.^162^ Enabling more witnesses (particularly those for the prosecution) to be competent to testify at trial, and assisting them to do so to the best of their ability, thus marks a significant development to enabling convictions.

Assisting the vulnerable to give quality evidence also promotes the principle of accurate fact-finding.^163^ This principle is described as ‘the ultimate golden thread tying criminal proceedings to the public interest’.^164^ It is a commitment to factual accuracy that ensures that verdicts represent more than ‘legal classifications’ so that they express ‘moral culpability for actual, factual, wrongdoing’.^165^ Factual accuracy is clearly jeopardised when cases against those who have offended against children or other vulnerable groups are routinely dismissed pre-trial, or acquitted at trial, due to restrictive evidence rules that negatively affect the ability of witnesses to testify.

Ensuring that vulnerable individuals can gain access to the criminal justice system and participate in it fully is important for the maintenance of public confidence in the administration of criminal justice.^166^ Packer highlights that the repression of criminal conduct is the key purpose underpinning both the crime control and due process models of criminal justice.^167^ The state’s ability to protect citizens from criminal wrongdoing and to bring offenders to justice is thus a vital component for public confidence in the system.^168^ The system’s historic failure to protect children and other vulnerable groups from crime thus undermines this key principle of criminal justice.

The principle of humane treatment also plays an important role in underpinning the instrumental aim of improving evidence quality. This operates in two ways: (i) enabling vulnerable people to access the criminal justice system and give admissible evidence in trials *per se*; and (2) enhancing the ability of a vulnerable person to give good-quality evidence in the trial setting.

On these points, the criminal justice system is the means through which the state remedies criminal violations of a person’s autonomy and other criminal wrongdoing. The ability of vulnerable persons to report crime, to have those they accuse prosecuted, and to testify in court against them in order to secure a conviction are matters of humane treatment. So too is the ability of a vulnerable defendant to testify in their defence should they wish to do so. A failure to enable vulnerable people to access the criminal justice system and to give their best evidence amounts to a systemic failure to protect vulnerable individuals from crime and abuse more generally. Absent suitable adaptations, the people who are the most vulnerable to victimisation (or to false accusations), such as the learning disabled^169^ and the young, are left increasingly vulnerable due to the system’s inability to cater for them. This exclusion is likely to cause a heightened amount of suffering for the individual concerned.

Parliament discussed the ability of vulnerable people to give evidence in criminal trials as a key element of ‘access to justice’ for the most vulnerable in society^170^ when debating the special measures provisions. Lord Williams stated that ‘Far too many cases involving the most vulnerable victims have been abandoned, or not even begun, because the victim cannot adequately give his side of the story’.^171^ This was echoed by others, including Lord Rix, who highlighted that ‘It is a basic tenet of civil society that people whose rights have been infringed should be able to secure justice through the judicial process. That has not been happening.’^172^

It is argued, therefore, that to exclude vulnerable individuals from accessing the criminal justice system, or from meaningful participation within it, is inherently inhumane. It is as a function of the principle of humane treatment, then, that adaptations are available to vulnerable and intimidated witnesses in order that they are able to access the criminal justice system at all, and to give their best evidence within it. This ensures that alleged victims can seek remedy for wrongdoing against them and those accused of offences are able to testify in their defence if they so wish.

#### (ii) Humane treatment as a way of achieving the instrumental goal of best evidence

As well as providing a solid basis for the need to improve access to the system and the quality of evidence elicited from vulnerable witnesses and defendants, promoting humane treatment also helps to achieve the instrumental goal of best evidence. This is because a correlation exists between the way a person is treated within the process and the resulting completeness, coherence and accuracy of their evidence.^173^ The traditional adversarial rules requiring witnesses to give evidence orally, in court, in front of the accused, often some months or even years after the alleged incident about which they are testifying, often cause stress and trauma for the witness.^174^ A consequence of this treatment is a potential diminution in the quality of evidence, since distressed witnesses are often confused, emotional, impressionable and forgetful.

The provision of special measures such as screens or live links can improve evidence quality by neutralising some of those elements of the criminal process that may cause a witness to feel distressed.^175^ Special measures essentially enhance evidence quality by improving the treatment of a vulnerable or intimidated witness in the trial. Treating the vulnerable humanely is, therefore, a method through which the quality of their evidence is improved. This means that when special measures are secured on evidence quality grounds as per the YJCEA, their use often serves to simultaneously improve the treatment of the vulnerable and intimidated as they participate in the process. In this vein, the nature of the relationship between the instrumental goal of evidence quality and deontological value of humane treatment is mutually reinforcing.

There are three caveats to this. The first is that we cannot blindly assume that the use of special measures will always be the most humane way forward. For instance, some witnesses find that watching their ‘achieving best evidence’ interview—which is played in lieu of live evidence-in-chief—is distressing in itself.^176^ It may be, therefore, that a measure that improves evidence quality can also result in heightened suffering at a different point in the process. The second caveat is that we should take care not to view special measures as a ‘fix-all’ solution. It may sometimes be the case that a person’s vulnerability or intimidation is so grave that the use of special measures does not address the heightened suffering that giving evidence in a criminal trial would cause. The only humane solution here may be that they do not give evidence at all,^177^ or, in the case of intimidation, that anonymous witness provisions^178^ or hearsay statements^179^ are used.

The third caveat is that this analysis is based on an assumption that there is agreement among legislators and the legal profession as to what constitutes ‘best evidence’ or evidence of good quality. In fact, there is evidence to suggest that this is not the case, and that many advocates actually consider best evidence to be that which is live, emotional, and raw because of its perceived impact on the jury.^180^ With this in mind, even where a vulnerable or intimidated witness or defendant might technically qualify for special measures (where evidence quality is conceived of in terms of its completeness, coherence and accuracy), some practitioners might not invoke special measures at all. Even if they do so, they may opt for screens instead of live link, to maximise the perceived impact of the evidence.^181^ In such instances, eligibility for and the use of special measures cannot be assumed to simultaneously promote the humane treatment of a vulnerable or intimidated witness or defendant.

#### (iii) What if evidence quality is not an issue?

The final aspect of the relationship between evidence quality and humane treatment that requires consideration is the occasions where a witness or defendant is vulnerable or intimidated (as per part one of the eligibility test—see Figure 1) but it is concluded that the quality of their evidence will not be diminished as a result. In this instance, a vulnerable or intimidated witness/defendant is not eligible for special measures. It is here that the so-called ‘mutually reinforcing’ relationship between evidence quality and humane treatment comes unstuck. In circumstances where evidence quality is not perceived as under threat, it is fallacious to assume the same of humane treatment. Absent special measures, the treatment of a vulnerable or intimidated witness or defendant—required to give evidence live and in open court—may not meet the standards required for humane treatment.

As the law is currently constructed, there are no grounds of eligibility to secure special measures solely to foster humane treatment. This means that there is currently a gap in the provision of special measures to the vulnerable or intimidated when evidence quality is not a concern, which could jeopardise the state’s fulfilment of the principle of humane treatment. The goal of humane treatment is thus one that operates separately to, as well as in tandem with, the goal of improving evidence quality.

The protection of the principle of humane treatment should thus be a stand-alone criterion for the provision of special measures to vulnerable and intimidated witnesses. This is in line with process-based theories of procedural justice, concerned with securing procedures that treat individuals fairly irrespective of any instrumental advantage that might ensue.^182^ It is thus about more than the use of reliable procedures to attain factually accurate verdicts; it is about ensuring that individuals are treated as moral equals and shown adequate respect in the process.^183^ A relational approach to procedural justice requires that the state adopts ‘procedures that convey the message that they are impartial, trustworthy, respectful, and willing to listen’,^184^ as well as demonstrating a concern for the welfare of those appearing before them.^185^ The principle of humane treatment should, therefore, be understood as an articulation of this broader goal of procedural justice: to ensure that witnesses and those accused of criminal offences are not treated as objects of state control, but as subjects worthy of dignity and respect. As Roberts and Zuckerman note, ‘shabby treatment of witnesses is objectionable *per se*, and may engage the state’s duty of humane treatment if apparently tolerated and allowed to continue without restraint, sanction, or redress’.^186^

The power dynamic between the state and other individuals in the adversarial criminal trial requires the state to act to protect lay participants from harm.^187^ The compellability of witnesses^188^ (other than the accused) to give evidence places an extra burden of responsibility on the state to ensure that they are treated humanely in the process, and not subjected to heightened suffering compared to that which is inherent within the adversarial process. The position of the accused, as a party to proceedings brought by and against the state, also heightens the duty on the state to ensure that it treats the accused humanely. While the accused is not strictly compellable as a witness, the erosion of the right to silence^189^ and evidential presumptions that require comment from the accused^190^ will often *de facto* compel the accused to testify in their defence. The provision of special measures to vulnerable or intimidated witnesses and accused persons on the sole grounds of humane treatment can at least partially discharge the state’s duty of humane treatment.

It is conceded that the demand for special measures in such circumstances may be rare, due to the knowledge that intense stress or trauma when giving evidence is in turn likely to diminish the evidence’s quality. This link between these factors is likely to mean that, in practice, special measures are usually secured when a witness is vulnerable or intimidated in order to enhance evidence quality.^191^ However, it is not beyond the bounds of possibility that a lawyer or judge will consider that a person’s vulnerability or intimidation will not diminish the quality of their evidence, and thus deny special measures. The rarity with which this might occur does not justify the lack of legal grounds for protecting *all* vulnerable or intimidated persons involved in testifying in a criminal trial from inhumane treatment.

Indeed, the possibility of this gap in eligibility was considered in Parliament in the context of discussions of the importance of the court giving reasons for refusing applications ‘if a court accepts that a witness has a disability but does not accept that special measures would improve the quality of that evidence’.^192^ This was also identified in the House of Commons by Mr Paul Boateng (Minister of State, Home Office) noting that ‘not all physical or mental disorders or disabilities affect a witness’s ability to give his best evidence’.^193^ What this means in practice is that a witness or defendants will not be afforded any adaptation to the proceedings if it is concluded that the quality of their evidence will not be affected (or that even if it will, none of the available special measures are thought appropriate to counteract it). This is so even if such an individual has (for example) an anxiety disorder or a deformative physical disability, or is in fear or distress in connection with testifying in the proceedings. It is certainly feasible that the prospect and eventuality of testifying without adaptation or assistance for such individuals would be stressful to an extent that amounts to heightened suffering and is thus inhumane. It is also clear that the use of a special measure to keep them out of the courtroom, or out of sight of the accused/the public gallery, could allay some of this distress and help them to feel more at ease. In practice, it may be the case that such individuals are granted special measures anyway, despite the absence of formal legal grounds. However, it is in the interests of consistency and fairness that this is available on a sound legal basis and not left to the discretion of particularly humane trial judges/counsel.

## 5. Conclusion

This article has shown that two key sets of concerns underpinned the development of special measures provision to vulnerable and intimidated witnesses. These related (i) to the (in)humane treatment of such individuals and (ii) to the (poor) quality of evidence elicited in criminal trials and the difficulties this created in securing convictions. The ensuing YJCEA awards eligibility for special measures to vulnerable and intimidated witnesses solely on the grounds of enhancing/protecting the quality of their evidence. There is a distinct absence of statutory (or other) grounds for providing special measures to improve the treatment of vulnerable and intimidated witnesses in the course of their testimony. This is in direct contradiction with the policy recommendations made to the government on this issue. The same is true for vulnerable or intimidated defendants.

The principle of humane treatment is conceived of as a positive obligation on the state to protect those involved in criminal trials from inhumane treatment, which is defined in this article as heightened suffering beyond that which is inherent and accepted in the adversarial context. In other words, it is a level of suffering which is disproportionate to the legitimate aim of accurate fact-finding. This might manifest as an unhealthy level of stress connected with the prospect of testifying in the proceedings, which could (but does not have to) result in sleepless nights and other altered behaviours and bodily functions. Its assessment must be contextualised, taking into account the specific characteristics and circumstances of the individual involved.

This article assesses whether the current legal provision of special measures, without direct reference to the humane treatment of vulnerable and intimidated witnesses or defendants, still sufficiently upholds (the principle of) humane treatment. This involves an analysis of the complex relationship between the instrumental goal of improving evidence quality and the deontological value of humane treatment. Cooper and Roberts’s assertion that the two are in a mutually reinforcing relationship is proved at least partially accurate. If special measures are secured on evidence quality grounds, it is likely that they will simultaneously improve the treatment of the individual concerned, rendering it more humane. However, where special measures are denied to someone who is otherwise vulnerable or intimidated, because it is not thought that they are required on the grounds of evidence quality, it cannot also be assumed that they are not needed on the grounds of humane treatment. There is thus a gap in the protection of vulnerable or intimidated individuals testifying in the criminal trial.

To remedy this, it is suggested that an additional ground of eligibility is added to the YJCEA for special measures. This would make special measures available to vulnerable or intimidated witnesses and accused persons where giving evidence is otherwise ‘likely to cause a significant or heightened amount of suffering’. This would make it similar to the Scottish provision of special measures, which has a specific eligibility criterion for witnesses who are at risk of harm due to giving evidence.^194^ It would also bring it in line with the special measures provisions as introduced into the Family Procedure Rules and Family Practice Directions, which require the consideration of special measures for vulnerable participants giving evidence where a party or witness would otherwise attend the hearing with ‘significant distress’.^195^ This approach is preferable to simply removing the evidence quality requirement from the current construction of the law and making eligibility automatic if the vulnerability/intimidation requirement is satisfied. This is because adding a ground of eligibility draws specific attention to the importance of protecting the vulnerable or intimidated from this type of harm as a proper and essential use of the available adaptations. This might prove especially useful to temper any practice where practitioners might seek to avoid the use of (particular) special measures on tactical grounds relating to beliefs about evidence quality and its perceived impact on jurors.

More broadly, the findings from this article tell us something about the implications of the tension between the instrumental and intrinsic value in other contexts. One such example would be the admissibility of evidence such as past sexual history evidence. Using special measures as an example, this article shows that focusing solely on instrumental issues can leave a gap in the protection of those involved in criminal proceedings. In order for the criminal justice system to maintain a consistent commitment to the principle of humane treatment, and thus for those within it to be fairly treated, the way in which evidential rules are constructed should have explicit regard to this deontological value.

## Footnotes

1 Youth Justice and Criminal Evidence Act 1999 (YJCEA), s 23, s 24, s 29 and s 30 respectively.

2 YJCEA, s 25, s 26, s 27 and s 28 respectively.

3 Criminal Practice Directions [2016] EWCA Crim 1714, 18A.2.

4 YJCEA, s 16 for non-accused witnesses and s 33A for the accused.

5 YJCEA, s 17(1).

6 YJCEA, s 17(2).

7 YJCEA, s 16(5).

8 YJCEA, s 16(1)(b), s 17(1).

9 For a critique of the prevailing inequality, see Samantha Fairclough, ‘Speaking Up for Injustice: Reconsidering the Provision of Special Measures through the Lens of Equality’ [2018] CLR 4.

10 Defendant witnesses are not compellable to give evidence as per YJCEA, s 53(4).

11 YJCEA, s 33A. This was a late insertion into the YJCEA via the Police and Justice Act 2006. The development of special measures for the accused is discussed in more depth in section 4.

12 See *C v Sevenoaks Youth Court* [2009] EWHC 3088 (Admin); *R v Waltham Forest Youth Court* [2004] EWHC 715 (Admin) [31]; Criminal Procedure Rules and Criminal Practice Directions [2016] EWCA Crim 1714.

13 Paul Roberts and Adrian Zuckerman, *Criminal Evidence* (2nd edn, OUP 2010) 18–19.

14 Lorraine Wolhunter, Neil Olley and David Denham, *Victimology: Victimisation and Victims’ Rights* (Routledge 2008) 161.

15 See CPS, *Safeguarding Children as Victims and Witnesses: Legal Guidance* (updated October 2019) <www.cps.gov.uk/legal-guidance/safeguarding-children-victims-and-witnesses>.

16 See Denise Mayerson and Catriona Mackenzie, ‘Procedural Justice and the Law’ (2018) 13 Philosophy Compass 1.

17 Prior to this, the courts had used their inherent power to make adjustments to the way some witnesses gave evidence, which we now recognise as special measures. For a comprehensive account of this, see Ruth Marchant, ‘Special Measures’ in Penny Cooper and Heather Norton (eds), *Vulnerable People and the Criminal Justice System* (OUP 2017) 333.

18 Though see Carol Hedderman, *Children’s Evidence: The Need for Corroboration* (1987) Home Office Research and Planning Unit Paper 41.

19 John Spencer, ‘Child Witnesses and Cross-Examination at Trial: Must It Continue?’ (2011) 3 Arch Rev 7.

20 See John Spencer and Michael Lamb (eds), *Children and Cross-Examination: Time to Change the Rules?* (Hart Publishing 2012) 2–4.

21 For a discussion of high-profile child abuse cases in which a defendant was acquitted or not even prosecuted, see John Spencer, ‘Section 28 YJCEA 1999 and Pre-trial Cross-Examination: Where We’ve Got to Now and How We Got There’ (2019) 4 Arch Rev 4.

22 Criminal Justice Act 1988, s 34 and later Criminal Justice and Public Order Act 1994 (CJPOA), s 32.

23 Criminal Justice Act 1988, s 32. It was automatically available to children under the age of 14 who were the alleged victim of (a) an assault, injury, threat of injury; (b) a cruelty offence; (c) a sexual offence; or (d) an offence for the assistance or encouragement of an offence falling into categories (a)–(c).

24 HL Deb 14 July 1987, vol 488, col 980.

25 HC Deb 27 November 1986, vol 106, col 469 (Mr Hurd).

26 ibid.

27 HC Deb 31 March 1987, vol 113, col 957 (Mr Mellor).

28 HC Deb 18 January 1988, vol 125, col 689 (Mr Hurd).

29 HL Deb 27 April 1987, vol 486, col 1318 (Lord Meston).

30 HC Deb 18 January 1988, vol 125, col 721 (Mr Tim Devlin).

31 HC Deb 20 June 1988, vol 135, col 871 (Dr Norman A Godman).

32 Lord Thomas Pigot, *Report of the Advisory Group on Video Evidence* (Home Office 1989) para 2.9 **(***Pigot Report*).

33 ibid para 2.14.

34 ibid para 2.18.

35 ibid para 2.18.

36 ibid para 2.38.

37 ibid para 3.5.

38 ibid para 3.8.

39 Criminal Justice Act 1988, s 32A, inserted by Criminal Justice Act 1991, s 54.

40 Children under 14 were eligible in respect of crimes relating to assault, injury or cruelty against them; children under 17 were eligible for sexual offences.

41 HC Deb 20 November 1990, vol 181, col 168 (Mr Lawrence).

42 HL Deb 12 March 1991, vol 527, col 136.

43 HL Deb 22 April 1991, vol 528, col 47 (Earl Russell).

44 See eg HL Deb 22 April 1991, vol 528, col 76.

45 HL Deb 22 April 1991, vol 528, col 33 (Lord Mottistone).

46 See *R v Hayes* [1977] 1 WLR 234 [237].

47 Andrew Sanders and others, *Victims with Learning Disabilities: Negotiating the Criminal Justice System* (1997) University of Oxford Centre for Criminological Research Occasional Paper No17, 39.

48 ibid 57.

49 ibid 8.

50 Andrew Sanders and others, *Witnesses with Learning Difficulties* (1996) Home Office Research and Statistics Directorate Research Findings No 44, 3.

51 Sanders and others, *Victims with Learning Disabilities* (n 47) 8.

52 ibid.

53 Home Office, *Speaking Up for Justice: Report of the Interdepartmental Working Group on the Treatment of Vulnerable or Intimidated Witnesses in the Criminal Justice System* (Home Office 1998) (*Speaking Up for Justice*).

54 ibid paras 1.1–1.2.

55 ibid Literature Review 105.

56 As well as amending the competency rules, as now enacted in YJCEA, s 53.

57
*Speaking Up for Justice* (n 53) para 3.29.

58 ibid para 3.29.

59 The exact format of this legislation is discussed in section 4.

60 For example, HC Deb 15 April 1999, vol 329, col 458.

61 HL Deb 2 March 1999, vol 597, col 1610 (Lord Williams).

62 As is discussed later in section 4, the humane treatment of vulnerable witnesses can also be conceived of as an instrumental way to improve evidence quality.

63 Evaluations of the law’s success at improving evidence quality can be found elsewhere. For an overview, see Samantha Fairclough, *Special Measures Literature Review* (Victims’ Commissioners Office 2020).

64 Along with those of accurate fact-finding, protection of the innocent, minimum state intervention, and upholding standards of moral propriety in criminal adjudication. See Roberts and Zuckerman (n 13) 18–19.

65 ibid 21.

66 ibid.

67 There is significant disagreement in the literature around the utility of dignity as a concept, and exactly what it does conceptually. See Lucy Michael, ‘Defining Dignity and Its Place in Human Rights’ (2015) 20(1) The New Bioethics 12. This article aligns with those, such as Kant, who see dignity as permanent, unconditional and inviolable, or in other words see humans as holding ‘full-inherent dignity’.

68 Immanuel Kant (1797) in Mary Gregor (ed), *The Metaphysics of Morals* (CUP 1996) 209.

69 Paul Roberts, ‘Subjects, Objects, and Values in Criminal Adjudication’ in Anthony Duff and others (eds), *The Trial on Trial. Volume 2. Judgment and Calling to Account* (Hart Publishing 2006) 43.

70 ibid 43–4.

71 ibid 44.

72 ibid 45.

73 ibid 45.

74 Linda Mulcahy, ‘Architects of Justice: The Politics of Courtroom Design’ (2007) Social and Legal Studies 383, 387.

75 Linda Mulcahy, *Legal Architecture: Justice, Due Process and the Place of Law* (Routledge 2011) 7–8. Doak similarly describes the courtroom as an ‘unfamiliar and forbidding environment’: Jonathan Doak, *Victims’ Rights, Human Rights and Criminal Justice: Reconceiving the Role of Third Parties* (Hart Publishing 2008) 51.

76 Doak (n 75) 51.

77 As the Court of Appeal has increasingly recognised since *R v Barker* [2010] EWCA Crim 4.

78 See Roberts and Zuckerman (n 13) 58, 293, 347.

79 This issue was touched upon in the case of *T v United Kingdom* App No 24724/94 (ECtHR, 16 December 1999). Lord Reed, in his concurring opinion, noted that for treatment to fall foul of art 3 ECHR the ‘suffering or humiliation must … go beyond that inevitable element of suffering or humiliation connected with a given form of legitimate treatment’ and further noted that ‘any trial is liable to cause mental suffering and feelings of humiliation … Nevertheless, a trial could not ordinarily be described as inhuman or degrading treatment, since it is a legitimate form of procedure according to prevailing standards’.

80 Though it could sometimes be inappropriate without reasonable adjustments.

81 In *T v United Kingdom* (n 79), Judge Pastor Ridruejo and others’ partly dissenting opinion highlighted an important qualification to Lord Reed’s previous point about inevitable suffering, noting that ‘there is no good reason to presume that the minimum level of suffering qualifying as ill-treatment [for art 3 ECHR] cannot be inflicted by a court exercising its lawful authority in the course of a trial’. This logic is similarly applicable with regards to inhumane treatment.

82 See also Mavronicola’s example of criminal punishment—punishment inflicts suffering by design, but when does that legitimate suffering go too far? Natasa Mavronicola, ‘Crime, Punishment and Article 3 ECHR: Puzzles and Prospects of Applying an Absolute Right in a Penal Context’ (2015) 15 Human Rights Law Review 721, 740.

83 There is not space in this article for a full discussion of this, but see further Michael (n 67) 26–8; Jeff Malpras, ‘Human Dignity and Human Being’ in Jeff Malpras and Norelle Lickiss (eds), *Perspectives on Human Dignity: A Conversation* (Springer 2007).

84 This ‘balancing exercise’ is akin to that which takes place when considering potential violations of art 8 ECHR, the right to respect for private and family life, which is qualified in situations where it is ‘in accordance with the law and is necessary in a democratic society in the interests of national security, public safety or the economic wellbeing of the country, for the prevention of disorder or crime, for the protection of health or morals, or for the protection of the rights and freedoms of others’ (see art 8(2)).

85 ECHR art 3.

86 Though inhuman and degrading might not strictly be in such a hierarchy but instead demark different forms of ill-treatment, see Mavronicola, ‘Crime, Punishment and Article 3 ECHR’ (n 82) 740, 725.

87
*Ireland v United Kingdom* App No 5310/71 (ECtHR, 18 January 1978).

88 ibid para 167.

89 ibid.

90 ibid.

91 ibid para 162.

92
*A v United Kingdom* App No 25599/94 (ECtHR, 23 September 1998) para 20.

93 See Jeremy Waldron, ‘Inhuman and Degrading Treatment: The Words Themselves’ (2010) 23(2) CJLJ 269, 278.

94 John Kleinig, ‘The Hardness of Hard Treatment’ in Andrew Ashworth and Martin Wasik (eds), *Fundamentals of Sentencing Theory* (Clarendon 1998) 285.

95
*Ireland v United Kingdom* (n 87) para 162.

96 Jeremy Waldron, ‘Torture and Positive Law: Jurisprudence for the White House’ (2005) 105(6) Colum L Rev 1681, 1745.

97
*Ireland v United Kingdom* (n 87).

98 ibid para 15 (Judge Fitzmaurice).

99 The case concerned the use of ‘five techniques’ during the interrogation of persons in detention in connection with terrorism offences. The ECtHR ruled, by 16 votes to one, that the treatment was inhuman as per art 3 ECHR. It should be noted here that the quality of his concrete finding in relation to the five techniques, which he considered not to have violated art 3, is very problematic. Indeed, in 2018, the ECtHR even considered whether it should revise its decision and find that the five techniques actually amounted to torture. See Alan Greene, ‘Ireland v the UK and the Hooded Men: A Missed Opportunity?’ (2018) <https://strasbourgobservers.com/2018/04/25/ireland-v-the-uk-and-the-hooded-men-a-missed-opportunity/>. With this said, his general point about maltreatment that follows is useful for conceptualising the principle of humane treatment.

100
*Ireland v United Kingdom* (n 87) para 22 (Judge Fitzmaurice).

101 Louise Ellison and Vanessa Munro, ‘Taking Trauma Seriously: Critical Reflections on the Criminal Justice Process’ (2017) 21 International Journal of Evidence and Proof 183, 184.

102 See the discussion of this notion of procedural equality as a key principle underpinning the special measures scheme in Fairclough (n 9).

103 Debbie Cooper and Paul Roberts, *Special Measures for Vulnerable and Intimidated Witnesses: An Analysis of Crown Prosecution Service Monitoring Data* (CPS 2005) 15.

104 Roberts and Zuckerman (n 13) 58–62.

105 There is not space for a full discussion of this here, but see Fairclough (n 9) 11–15.

106
*T v United Kingdom* (n 79).

107 The applicant asked the ECtHR to find violations of art 3 ECHR in respect of his trial and sentence, art 14 in respect of the alleged discriminatory attribution to him of criminal responsibility, art 6(1) in respect of his trial and the tariff-setting procedure, art 5(1) in respect of the sentence of detention and art 5(4) in respect of the absence of any judicial review of the continuing legality of his detention.

108
*T v United Kingdom* (n 79) para 56.

109 ibid para 61.

110 ibid para 17.

111 ibid para 77.

112 App No 41107/10 (ECtHR 28 May 2015).

113 ibid para 115.

114 In much the same way that it would not be fruitful to do so for other vague legal terms such as ‘torture’ or ‘public disorder’, see Tim Endicott, *Vagueness in the Law* (OUP 2000) 190.

115 Joseph Raz, ‘The Rule of Law and Its Virtue’ The Authority of Law: Essays on Law and Morality (OUP 1979).

116 Lon Fuller, *The Morality of Law* (Yale UP 1969).

117 See Natasa Mavronicola, ‘What Is an Absolute Right?: Deciphering Absoluteness in the Context of Article 3 of the European Convention on Human Rights’ (2012) 12 Human Rights Law Review 723.

118 Endicott argues that the creation of sharp ‘boundaries’ for vague terms is problematic: Endicott (n 114) ch 6.

119 See *Interim Report of the Special Rapporteur on Torture and Other Cruel, Inhuman or Degrading Punishment* (July 2018) United Nations General Assembly Report A/73/150, para 43, which states that where there is uncertainty as to the precise scope of the prohibition of torture and ill-treatment, ‘States should always err on the side of caution’.

120 Endicott discusses this in the context of time limits for trials under art 6 ECHR. Article 6 provides a right to trial on criminal charges ‘within a reasonable time’—a vague standard. Endicott notes that by not giving the prosecution a deadline, the Convention gives them a reason to act as soon as they are able to, both to avoid the delay being seen as unreasonable, and so that they have reasons as to why they have taken the time they have should it become an issue. See Endicott (n 114) 203.

121 Stress symptoms taken from <www.mentalhealth.org.uk/a-to-z/s/stress>.

122 Sheldon Cohen and others, ‘A Global Measure of Perceived Stress’ (1983) 24 Journal of Health and Social Behaviour 385. It is one of the most widely disseminated methods of assessing psychological stress, as per Miguel Vallejo and others, ‘Determining Factors for Stress Perception Assessed with the Perceived Stress Scale (PSS-4) in Spanish and Other European Samples’ (2018) 9(37) Frontiers in Psychology 1.

123 Charles Spielberger and others, *Manual for the State-Trait Anxiety Inventory* (Consulting Psychologists Press 1983). It is often used in research to measure caregiver distress. See eg Jennifer Greene and others, ‘The Relationship between Family Caregiving and the Mental Health of Emerging Young Adult Caregivers’ (2017) 44 *Journal of Behavioural Health Services and Research* 551.

124 eg ‘I am tense’ or ‘I am worried’.

125 eg ‘I worry too much over something that really doesn’t matter’.

126 These suggestions should be understood as tentative given that I am not an expert in the understanding and measurement of stress. Collaboration with psychologists is needed to test the viability of these (and other) options. This would also require work to try to establish the ‘normal’ and acceptable level of stress we might expect to see among those testifying in the trial and when these levels are exceeded so that a witness or defendant’s participation (absent adaptations) becomes inhumane.

127 In the language of the ECHR, the principle of humane treatment thus amounts to something of a positive obligation to avoid inhumane treatment.

128 This discussion is too large to have here. The vulnerable and intimidated criteria can be criticised on the grounds of being over- and under-inclusive. Notably, Fineman’s vulnerability theory would reject the notion that only some people in defined categories are vulnerable, instead favouring the approach that we are all vulnerable by virtue of our embodiment and embeddedness in social, economic and political institutions. See Martha Fineman, ‘The Vulnerable Subject: Anchoring Equality in the Human Condition’ (2008) 20 Yale JL & Feminism 1.

129 Under YJCEA, s 53, all persons, regardless of age, are presumed competent to give evidence so long as they can understand questions put to them and give answers that can be understood. In determining this, the court must treat the witness as having the benefit of any special measures available (s 55(3)).

130 As per *R v Pritchard* (1836) 7 C & P 303.

131 Problems with the identification of vulnerability are well documented. See Sanders and others, *Victims with Learning Disabilities* (n 47); Jessica Jacobson, *No-one Knows: Police Responses to Suspects Learning Disabilities and Difficulties—A Review of Policy and Practice* (Prison Reform Trust 2008) 27–8; Lord Bradley, *The Bradley Report: Review of People with Mental Health Problems or Learning Disabilities in the Criminal Justice System* (Department of Health 2009) 20; Jessica Jacobson and Jenny Talbot, *Vulnerable Defendants and the Criminal Courts: A Review of Provision for Adults and Children* (Prison Reform Trust 2009) 5–6, 13–14; Ali Wigzell, Amy Kirby and Jessica Jacobson, ‘The Youth Proceedings Advocacy Review: Final Report’ (Institute for Criminal Policy Research 2015) 46.

132 Evidence from other studies indicates that this is often the case. For example, Hamlyn and others found that those using special measures were less likely to feel anxious or distressed than those not using them: Becky Hamlyn and others, *Are Special Measures Working? Evidence from Surveys of Vulnerable and Intimidated Witnesses* (2004) Home Office Research Study 283. See also Jessica Jacobson, Gillian Hunter and Amy Kirby, ‘Structured Mayhem: Personal Experiences of the Crown Court’ (Criminal Justice Alliance 2015) 19; Rosie McLeod and others, *Court Experiences of Adults with Mental Health Conditions, Learning Disabilities and Limited Mental Capacity Report 1: Overview and Recommendations* (2010) Ministry of Justice Research Series 8/10, 24.

133 Those under 18 at the time they are required to give evidence.

134 YJCEA, s 16(1)(a).

135 YJCEA, s 16(2).

136 YJCEA, s 16(1)(b).

137 YJCEA, s 16(5).

138 YJCEA, s 19(2)(a).

139 YJCEA, s 17(1).

140 Complainants of sexual offences are automatically entitled to special measures without satisfying the two additional stages listed below, as per YJCEA, s 17(4).

141 See YJCEA, s 17(2).

142 YJCEA, s 17(1).

143 YJCEA, s 19(2)(a).

144 Above n 53.

145 HL Deb 1 February 1999, vol 596, col 1325.

146 ibid.

147 See YJCEA, s 21(3).

148 Usually via a screen instead as per YJCEA, s 21(4A).

149 See YJCEA, s 21(4)(ba) (emphasis added). This has not always been the case. The primary rule dictated, with no discretion, that when a relevant recording was available it should be admitted as the child victim’s evidence in chief, followed with cross-examination by live link. The Act was amended (Coroners and Justice Act 2009, s 100) and now the child’s views should be taken into account.

150 YJCEA, s 17(1).

151 YJCEA, s 22A(9) (emphasis added).

152 For reasons for the accused’s exclusion, see *Speaking Up for Justice* (n 53) para 3.28; for a damning appraisal of their validity, see Fairclough (n 9) 11–16.

153 On the issue of whether the legal source of special measures to the accused should be afforded any significance, see Samantha Fairclough, ‘The Consequences of Unenthusiastic Criminal Justice Reform: A Special Measures Case Study’ (2021) 21(2) Criminology and Criminal Justice 151.

154 YJCEA, s 33A. It is an inferior provision in comparison with that available to non-defendant witnesses. For example, it is not available to those with physical disabilities or who are ‘intimidated’ (in fear or distress in connection with testifying in the proceedings). For full discussion of the disparity, see Samantha Fairclough, ‘“It Doesn’t Happen … and I’ve Never Thought It Was Necessary For It To Happen”: Barriers to Defendants Giving Evidence by Live Link in Crown Court Trial’ (2017) 21 International Journal of Evidence and Proof 203.

155 YJCEA, s 33A(5).

156 YJCEA, s 33A(4)(a).

157 Criminal Practice Directions [2016] EWCA Crim 1714, CPD 3D.2; for a discussion, see Fairclough (n 9).

158 Cooper and Roberts (n 103) 166.

159 ibid.

160 ibid.

161 Helen Fenwick, ‘Procedural “Rights” of Victims of Crime: Public or Private Ordering of the Criminal Justice Process’ (1997) 60 MLR 317, 320.

162 The title of the CPS paper ‘No Witness, No Justice’ highlights the importance of witnesses to securing justice. See CPS, ‘No Witness, No Justice (NWNJ) Pilot Evaluation—Final Report’ (Criminal Justice System 2004).

163 This is the first of Roberts and Zuckerman’s five foundational principles of criminal evidence. See Roberts and Zuckerman (n 13) 18.

164 See ibid 19.

165 ibid.

166 Sanders, Young and Burton identify this as one of the goals of justice in Andrew Sanders, Richard Young and Mandy Burton, *Criminal Justice* (4th edn, OUP 2010) 47.

167 Herbert Packer, ‘Two Models of the Criminal Process’ (1964) 113 U Pa L Rev 1.

168 Roberts and Zuckerman (n 13) 17.

169 A Victim Support study highlights that people with learning disabilities are almost 3.5 times more likely to suffer serious violence, and 1.5 times more likely to be a victim of theft. See Polly Rossetti, Tamar Dinisman and Ania Moroz, ‘Insight Report: An Easy Target? Risk Factors Affecting Victimisation Rates for Violent Crime and Theft’ (Victim Support 2016) 4.

170 HL Deb 15 December 1998, vol 595, col 1240 (Lord Williams). See also HC Standing Committee E (5th sitting) 17 June 1999.

171 HL Deb 15 December 1998, vol 595 col 1237.

172 HL Deb 15 December 1998, vol 595 col 1293.

173 Rabiya Majeed-Ariss and others, ‘“Could Do Better”: Report on the Use of Special Measures in Sexual Offences Cases’ [2019] Criminology and Criminal Justice 3; Elizabeth Spruin and others, ‘Exploring the Impact of Specially Trained Dogs on the Court Experiences of Sexual Offence Survivors in England and Wales: An Exploratory Case Study’ [2019] European Journal on Criminal Policy and Research 2.

174 Spencer, ‘Section 28 YJCEA 1999 and Pre-trial Cross-Examination (n 21); Ellison and Munro (n 101) 184.

175 See Hamlyn and others (n 132); Jacobson, Hunter and Kirby (n 132) 19; McLeod and others (n 132) 24.

176 Joyce Plotnikoff and Richard Woolfson, ‘Falling Short?: A Snapshot of Young Witness Policy and Practice’ (NSPCC 2019) 399; Helen Beckett and Camille Warrington, ‘Making Justice Work: Experiences of Criminal Justice for Children and Young People Affected by Sexual Exploitation as Victims and Witnesses’ (University of Bedfordshire 2015) 35.

177 If they are the accused, then a direction as per CJPOA, s 35 could be warranted here, that hearing from a defendant suffering a ‘physical or mental condition’ would have been ‘undesirable’ and so no adverse inference should be drawn from their silence. However, questions around such a defendant’s fitness to plead and ability to effectively participate under art 6 YJCEA should also be asked.

178 See Coroners and Justice Act 2009, ss 86–90.

179 See Criminal Justice Act 2003, s 116(2)(e).

180 Mandy Burton and others, ‘Protecting Children in Criminal Proceedings: Parity for Child Witnesses and Child Defendants’ (2006) 18 Child and Family Law Quarterly 397, 404; Criminal Justice Joint Inspection, ‘Joint Inspection Report on the Experience of Young Victims and Witnesses in the Criminal Justice System’ (HMIC 2014) para 9.16; Samantha Fairclough, ‘Using Hawkins’ Surround, Field and Frames Concepts to Understand the Complexities of Special Measures Decision-Making in Crown Court Trials’ (2018) 45 Journal of Law and Society 457. Munro’s (2018) review of the evidence did not find any clear support for the view that the special measure used affects jury perceptions or criminal justice outcomes: see Vanessa Munro, *The Impact of the Use of Pre-recorded Evidence on Juror Decision-Making: An Evidence Review* (Scottish Government 2018).

181 Rabiya Majeed-Ariss and others (n 173) 12–13.

182 See Mayerson and Mackenzie (n 16).

183 ibid 9.

184 ibid 8.

185 ibid 9.

186 Roberts and Zuckerman (n 13) 348.

187 As Mavronicola argues in the context of state punishment: Mavronicola, ‘Crime, Punishment and Article 3 ECHR’ (n 82) 740.

188 YJCEA, s 53.

189 CJPOA, ss 34–8.

190 See eg Sexual Offences Act 2003, s 75.

191 While this may be true for witnesses eligible under the YJCEA scheme, the limited provision of special measures to the accused and the more onerous eligibility criteria mean that the same is not necessarily true of the accused, who may not even be able to secure special measures on evidence quality grounds.

192 HL Deb 1 February 1999, vol 596, col 1333 (Lord Williams).

193 HC Standing Committee E (5th sitting), 17 June 1999.

194 The Criminal Procedure (Scotland) Act 1995, s 271 defines vulnerable witnesses as (a) those who are under 18; (b) whose evidence is at risk of being diminished due to a mental disorder or fear or distress in connection with testifying in the proceedings; (c) who are the complainant of offences including sexual offences, trafficking offences, domestic abuse or stalking; or (d) who are considered at a significant risk of harm by reason of the fact that they are giving or due to give evidence in the proceedings. A dearth of empirical evidence on the use and effectiveness of special measures in Scotland means it is not clear how this plays out in practice and what, if any, difference it makes in terms of eligibility and the resulting treatment of vulnerable individuals.

195 Family Practice Direction 3AA, Vulnerable Persons: Participation in Proceedings and Giving Evidence, 2017, para 3.1(d) (Guidance about vulnerability: rule 3A.3(2) FPR).

